# Multi-platform omics analysis reveals molecular signature for COVID-19 pathogenesis, prognosis and drug target discovery

**DOI:** 10.1038/s41392-021-00508-4

**Published:** 2021-04-15

**Authors:** Yuming Li, Guixue Hou, Haibo Zhou, Yanqun Wang, Hein Min Tun, Airu Zhu, Jingxian Zhao, Fei Xiao, Shanwen Lin, Dongdong Liu, Dunrong Zhou, Lang Mai, Lu Zhang, Zhaoyong Zhang, Lijun Kuang, Jiao Guan, Qiushi Chen, Liyan Wen, Yanjun Zhang, Jianfen Zhuo, Fang Li, Zhen Zhuang, Zhao Chen, Ling Luo, Donglan Liu, Chunke Chen, Mian Gan, Nanshan Zhong, Jincun Zhao, Yan Ren, Yonghao Xu

**Affiliations:** 1 State Key Laboratory of Respiratory Disease, National Clinical Research Center for Respiratory Disease, Guangzhou Institute of Respiratory Health, the First Affiliated Hospital of Guangzhou Medical University, Guangzhou, China; 2 BGI-Shenzhen, Shenzhen, China; 3 The Sixth Affiliated Hospital of Guangzhou Medical University, Qingyuan People’s Hospital, Qingyuan, Guangdong China; 4 HKU-Pasteur Research Pole, School of Public Health, Li Ka Shing Faculty of Medicine, The University of Hong Kong, Hong Kong SAR, China; 5 Department of Infectious Diseases, Guangdong Provincial Key Laboratory of Biomedical Imaging, Guangdong Provincial Engineering Research Center of Molecular Imaging, The Fifth Affiliated Hospital, Sun Yat-sen University, Zhuhai, Guangdong China; 6 Yangjiang People’s Hospital, Yangjiang, Guangdong China; 7 Yangjiang Center for Disease Control and Prevention, Yangjiang, Guangdong China; 8 Guangzhou Eighth People’s Hospital of Guangzhou Medical University, Guangzhou, Guangdong China; 9 Guangzhou Customs District Technology Center, Guangzhou, China

**Keywords:** Predictive markers, Predictive markers

## Abstract

Disease progression prediction and therapeutic drug target discovery for Coronavirus disease 2019 (COVID-19) are particularly important, as there is still no effective strategy for severe COVID-19 patient treatment. Herein, we performed multi-platform omics analysis of serial plasma and urine samples collected from patients during the course of COVID-19. Integrative analyses of these omics data revealed several potential therapeutic targets, such as ANXA1 and CLEC3B. Molecular changes in plasma indicated dysregulation of macrophage and suppression of T cell functions in severe patients compared to those in non-severe patients. Further, we chose 25 important molecular signatures as potential biomarkers for the prediction of disease severity. The prediction power was validated using corresponding urine samples and plasma samples from new COVID-19 patient cohort, with AUC reached to 0.904 and 0.988, respectively. In conclusion, our omics data proposed not only potential therapeutic targets, but also biomarkers for understanding the pathogenesis of severe COVID-19.

## Introduction

Coronavirus disease 2019 (COVID-19) is caused by severe acute respiratory syndrome coronavirus 2 (SARS-CoV-2). As of January 19, 2021, over 93,805,612 confirmed cases with 2,026,093 deaths (mortality rate 2.16%) worldwide were reported to World Health Organization (WHO).^1^ Its most frequent clinical symptoms are pneumonia with fever cough and dyspnea. The severity rate of COVID-19 varies slightly worldwide ranged from 5 to 20%. As in New York (USA), 1151 patients (20%) required mechanical ventilation (severe case).^2^ In Italy, the proportion of intensive care unit (ICU) admissions were between 5 and 12% of the total COVID-19 cases.^3^ Based on the largest cohort study from China CDC, among 44,415 COVID-19 patients in China, 14% (6188 cases) were severe and 5% (2087) were critical.^4^ The mortality rate in patients who required mechanical ventilation (severe) reaches up to 88.1%, which is much higher compared with patients who did not receive mechanical ventilation (non-severe, mortality rate 11.7%).^2^

Compared with the non-severe cases, significantly higher concentrations of inflammatory cytokines (IL-6, IL-7, IL-10, IL-18, G-CSF, M-CSF, and MCP-1) were observed in the plasma of severe cases.^5^ In addition, total lymphocyte count, including CD4^+^ T cells, CD8^+^ T cells, B cells, and NK cells were significantly decreased in severe COVID-19 cases compared to non-severe cases, indicating dysregulation of immune responses.^6^ Autopsy studies of fatal cases revealed severe interstitial pneumonia of patients’ lungs with diffuse alveolar damage, as determined by the presence of hyaline membranes, interstitial thickening, vascular congestion, and inflammatory cell infiltration and polarized pulmonary macrophages.^7,8^

So far, there are no approved drugs available for COVID-19 treatment, although some drugs exhibiting antiviral activities in vitro were used to treat patients, including remdesivir and chloroquine.^9^ Most of the standard treatments for severe cases are supportive measures, such as mechanical ventilation and prevention of secondary infections.^10^ Therefore, it is critical to gain insights into molecular and metabolic changes in body fluids of COVID-19 patients, which will benefit drug discovery, patient treatment, as well as prognosis.

Omics analysis has been proved to efficiently identify drug targets or biomarkers for predicting the severity and progression of infectious diseases. In dengue hemorrhagic fever, eight candidate drugs targeting five proteins (ACTG1, CALR, ERC1, HSPA5, and SYNE2) were identified by multiple omics analysis, and five of these drugs (containing valparoic acid, sirolimus, resveratrol, vorinostat, and Y-27632) had been reported as effective treatments for flavivirus infection-induced diseases.^11^ Recently, proteome and metabolome techniques were used to explore molecular signatures of severe COVID-19 patients in plasma.^12^ However, in this study, most of thebiomarkers (22 proteins and 7 metabolites) identified in severe COVID-19 patients were proteins with limited clinical application due to the complicated and low-throughput protein quantification technology using liquid chromatography–mass spectrometry (LC–MS) platform. In addition, the metabolome analysis of this study primarily identified polar metabolites and the coverage of lipid classes was limited, which might lead to miss of important information as lipids play important roles during virus infection.^13^ Considering these limitations, further intensive studies are required for understanding the pathogenesis of SARS-CoV-2 infection, revealing molecular signatures to predict non-severe to severe transition in patients’ plasma or urine, and exploring potential drug targets for COVID-19 therapy.

In this study, we quantified proteins, amino acids, and lipids in plasma and urine samples from severe and non-severe COVID-19 patients, using healthy donors as controls using LC–MS technologies. For plasma proteins, small proteins were selectivity enriched and quantified by data independent acquisition (DIA) technology, which provided relatively deeper and complementary proteome data compared to previous study.^12^ Based on this strategy, unique protein signatures were discovered, such as tetranectin (TETN, CLEC3B) and cathelicidin antimicrobial peptide (CAMP), which could be used as new potential drug targets or biomarkers for COVID-19. As lipidomes technique was proved to have the potential to identify severe cases, lipid classes were further quantified by high-coverage and selectivity technology. Finally, 25 molecular signatures consist of 4 proteins and 21 lipids were defined as biomarker panel for disease prognosis, which could be a more feasible solution for prognosis and identification of severe patients, since sample preparation for lipid quantification is easier that will provide faster quantification for clinical application.

## Results

### Study design and patient cohorts

Serial blood and urine samples were collected from RT-PCR confirmed COVID-19 patients from four different hospitals (Supplementary Table 1 and Supplementary Dataset 1). In total, 27 blood samples and 19 urine samples from 15 severe patients, and 19 blood samples and 16 urine samples from 15 non-severe patients were obtained. According to the Chinese Government Diagnosis and Treatment Guideline (Trial seventh version), COVID-19 patients were classified into four subgroups based on their different clinical manifestations: (1) mild: mild clinical feature and no pneumonia symptoms; (2) common: fever, respiratory tract symptoms, and imaging features of pneumonia; (3) severe: respiratory distress and respiratory rate ≥30 times/min OR means oxygen saturation ≤93% in resting state OR arterial blood oxygen partial pressure (PaO_2_)/oxygen concentration (FiO_2_) ≤300 mmHg (1 mmHg = 0.133 kPa); and (4) critical illness: respiratory failure and require mechanical ventilation OR shock incidence OR require ICU care. In this study, all patients were divided into two subgroups, non-severe (mild and common) and severe (severe and critical illness) cohorts.

**Fig. 1:**
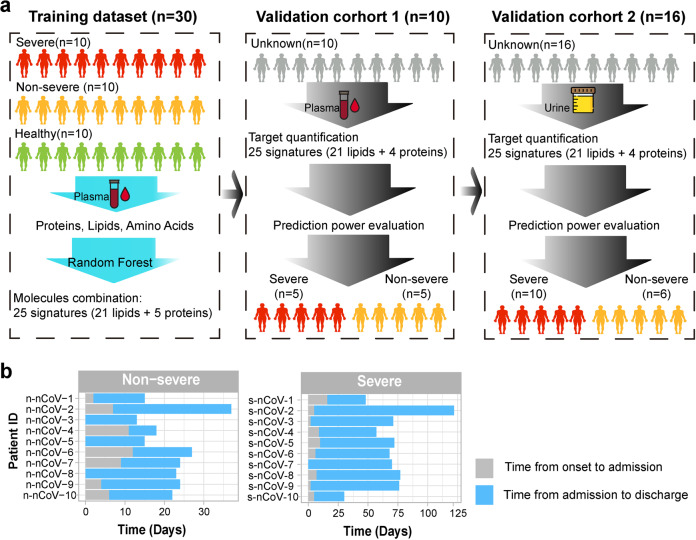
Overview of samples for multi-omics study. **a** Multi-omics analysis design with three datasets. The training dataset combined with severe, non-severe, and healthy controls, proteins, lipids, and amino acids were quantified in plasma and used for biomarker discovery, using random forest. The validation cohort 1 contained ten plasma samples from from non-severe and five severe patients, 25 molecules were targeted quantified for prediction evaluation. The validation cohort 2 contained urine samples corresponding to plasma samples in the training dataset, and prediction precision was further evaluated using targeted quantification. **b** Sample information of COVID-19 patients in the training dataset with time annotation from onset of disease to admission or from admission to discharge

Ten non-severe patients, ten severe patients, and ten healthy volunteers were included in plasma analysis as the training dataset. Proteins, amino acids, and lipids were extracted from plasma samples, and quantified, using untargeted profiling strategies by LC–MS platform. Signature molecules were selected for validation in the validation cohorts 1 and 2 by random forest algorithm. The validation cohort 1 contained ten new plasma samples acquired from an independent cohort of ten patients (containing five non-severe and five severe patients), and the corresponding urine samples acquired from patients of the training cohort were defined as the validation cohort 2, containing 35 serial samples acquired from six non-severe and ten severe patients. Potential biomarkers discovered in the training cohort were targeted, quantified, and used to evaluate the accuracy of predication in the validation cohorts. The whole design of these three cohorts was depicted in Fig. 1a. The detailed sampling information for proteome, amino acids, and lipidome in plasma and urines were illustrated in Supplementary Fig. 1.

Time points (days) of sample collection for the training dataset from onset of disease to admission and from admission to discharge was summarized in Fig. 1b. Ten blood samples from ten healthy donors were also collected as control. Detailed demographics and baseline characteristics were provided in Supplementary Table 1 and Supplementary Dataset 1. Compared to non-severe patients, severe patients showed significantly decreased lymphocyte count and frequency, as well as increased neutrophil and monocyte counts and frequencies (Supplementary Fig. 2), indicating dysregulation of immune response in severe COVID-19 patients.

### Plasma proteome, amino acids, and lipidome profiles of COVID-19 patients

**Fig. 2:**
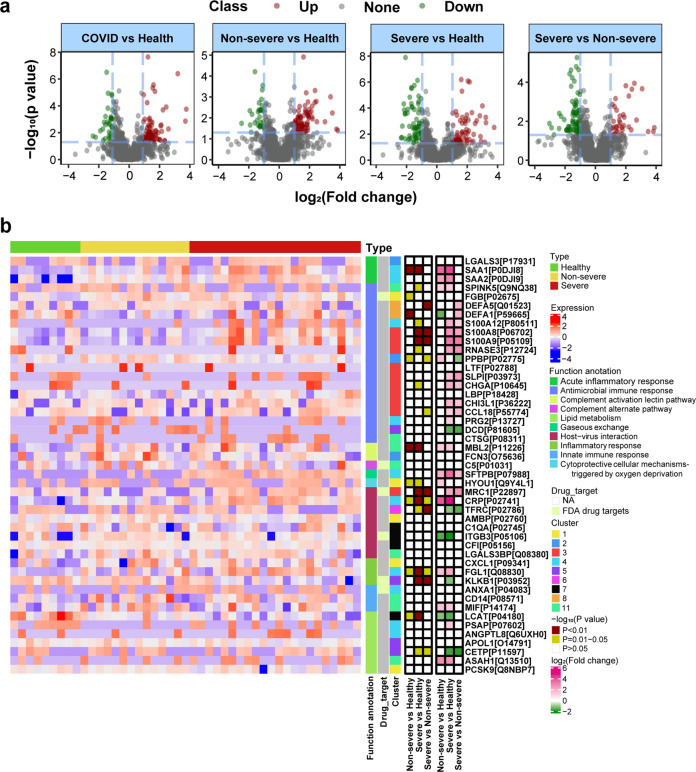
Proteome profiling of COVID-19 patients. **a** Volcano plot of quantified proteins in COVID-19 vs healthy group, non-severe vs healthy group, severe vs healthy group, and severe vs non-severe group. **b** Heatmap of selected differential proteins expression levels and associated *P* values for COVID-19 patients annotated with functions and drug targets information. FC fold change

Differential expressed proteins between COVID-19 vs healthy, non-severe vs healthy, severe vs healthy, and severe vs non-severe groups were explored. There were 1254 proteins quantified in total samples. Volcano plots in Fig. 2a revealed that 118 dysregulated proteins (86 upregulated and 32 downregulated) between the COVID-19 (non-severe and severe) and healthy group (pathway annotated in Supplementary Fig. 3a); 104 dysregulated proteins (76 upregulated and 28 downregulated) between the non-severe and healthy group (pathway annotated in Supplementary Fig. 3b); 143 dysregulated proteins (80 upregulated and 63 downregulated) between the severe and healthy group (pathway annotated in Supplementary Fig. 3c); and 105 dysregulated proteins (34 upregulated and 71 downregulated) between the severe and non-severe group (pathway annotated in Supplementary Fig. 3d) were identified. Pathway annotation for these differential proteins showed that they were enriched in immune and infectious pathways. The overlapped differential proteins defined in above comparisons were shown in Supplementary Fig. 4a. Furthermore, time-clustering analysis was used to further explore the protein expression patterns in health, non-severe to severe groups, which provided more indicators for understanding the infection of SARS-CoV-2. As shown in Supplementary Fig. 5, all quantified proteins could be clustered into 11 clusters according to their expression patterns. Proteins in cluster 3 (126 proteins), cluster 4 (117 proteins), and cluster 11 (119 proteins) were upregulated during infection (from non-severe to severe). Proteins in cluster 5 (116 proteins), cluster 7 (123 proteins), and cluster 10 (140 proteins) were downregulated during infection. To clarify these proteins selected by differential and time-cluster analysis, functional analysis was applied by searching their annotation in Human Protein Atlas (HPA)^14,15^ and uniprot.^16^ As shown in Fig. 2b, proteins related with SARS-CoV-2 infection were enriched in complement activation, inflammatory response, host–virus interaction, and lipid metabolism, such as chemokine C–C motif ligand 18 (CCL18), C-reactive protein (CRP), and cholesteryl ester transfer protein (CETP). CCL18 is highly expressed in lung tissues and has multiple functions in immune modulation.^16^ The increased expression of CCL18 in severe of COVID-19 patients compared with non-severe groups revealed that the activation of immune response during infection. The CRP is an acute inflammatory protein and plays important roles in host responses against viral infection,^17^ which was upregulated during SARS-CoV-2 infection. CETP is a plasma protein that facilitates the transportation of cholesteryl esters,^18^ which was decreased during SARS-CoV-2 infection compared with healthy controls. Transferring cholesterol to triglyceride-rich lipoproteins is an important step for the delivery of cholesterol to the liver. Therefore, decreased expression level of CETP could limit this transfer events and retard the RCT (reverse cholesterol transport) pathway, and lead to the accumulation of cholesterol in cells.^19^

**Fig. 3:**
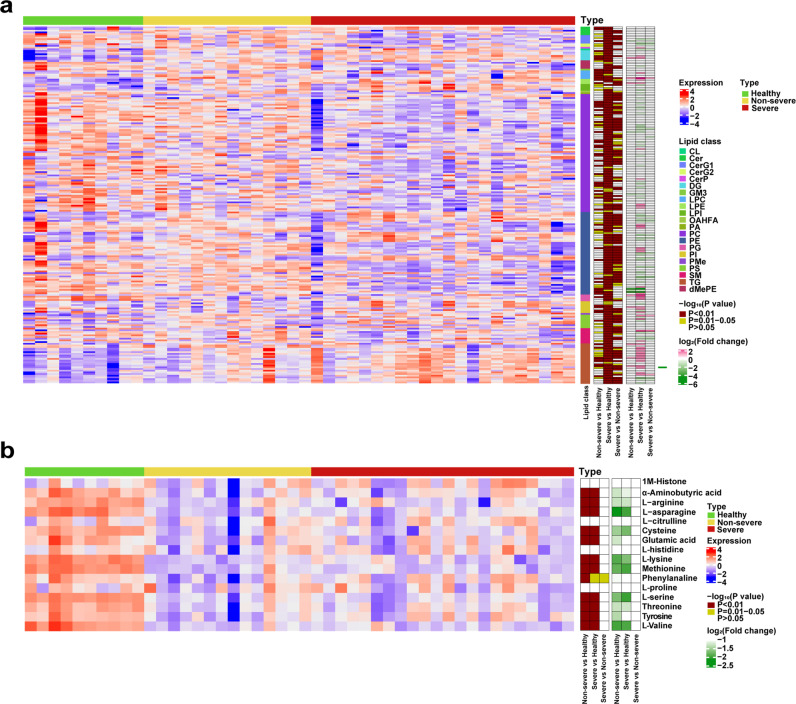
Heatmap of lipids and amino acids related with COVID-19. **a** Heatmap of lipids expression levels and associated *P* values for COVID-19 patients. FC fold change. **b** Heatmap of amino acids expression levels and associated *P* values for COVID-19 patients. FC fold change

Alterations in various plasma lipids were proved to be associated with inflammatory responses, such as those observed in sepsis and EBOV infection.^20^ Considering the aberrant protein profile related with lipids was identified in proteome analysis (Supplementary Fig. 3a–d), and the inhibition of lipid synthesis were proposed to affect COVID-19 disease pathogenesis. Therefore, lipid signatures related with SARS-CoV-2 infection were further explored. There were 664 lipid molecules quantified in all of the samples. Differential lipid analyses (Supplementary Fig. 4b and Supplementary Fig. 6) revealed the aberrant expression of lipid subclasses in non-severe and severe groups, as shown in Supplementary Fig. 6b–d. General signatures of SARS-CoV-2 infection primarily included increased lipids in phosphatidylinositols (PI), phosphoserine (PS), diacylglycerides, and triacylglycerides (TG), decreased lipids in phosphocholine (PC) and phosphoglycerol (PG), as illustrated in Supplementary Fig. 6a. Compared with the healthy group, the non-severe and severe groups shown increased PS and decreased PG, as shown in Supplementary Fig. 6b and Supplementary Fig. 6c. PS is a major component of procoagulant platelet microparticles,^21^ which is secreted by activated platelets. Increased PS levels in plasma in SARS-CoV-2 infection indicated increased platelet activation in these patients. Compared with healthy and non-severe groups, the abundance of glycosylceramide (CerG1) was decreased in severe group. CerG1 contains glucosylceramide (GlcCer), galactosylceramide (GalCer), and lactosylceramide in animals. GlcCer could activate protein C which can downregulate thrombin generation.^22^ Previous studies indicated that low plasma GlcCer levels were associated with the occurrence of venous thrombosis risk,^22^ which is common in severe patients infected with SARS-CoV-2. Besides, GalCer can be produced by bacteroides (a member of the human gut microbiome),^23^ which is known to downregulate ACE2 expression in murine gut, and bacteroides showed significant inverse correlation with fecal SARS-CoV-2 viral load in patients with COVID-19.^24^ Therefore, the decreased expression of CerG1 might reflect the reduction of bacteroides, which was correlated with the severity of COVID-19.

Time series cluster analysis using the expression patterns of lipids across healthy, non-severe, and severe groups could cluster these lipids into 11 clusters (Supplementary Fig. 7). Lipids showed continuously decreased levels of expression in cluster 1 (69 lipids), cluster 2 (91 lipids), and cluster 3 (69 lipids), while lipids in cluster 7 (59 lipids) and cluster 8 (87 lipids) were increased during infection, from healthy to non-severe, and from non-severe to severe syndrome. Heatmap in Fig. 3a for differential expressed lipids revealed the unique regulation pattern of lipids in plasma during SARS-CoV-2 infection.

Furthermore, 16 amino acids in plasma were quantified, because amino acids are vigorously interacted with lipids, such as serine with PS, which play important roles in infectious and inflammatory diseases.^25^ Herein, the concentrations of amino acids in healthy, non-severe, and severe groups with SARS-CoV-2 infection were compared. A prominent and acute reduction in plasma amino acids were observed in SARS-CoV-2-infected groups (Fig. 3b). Amino acids (e.g., glutamine) are consumed in inflammatory states to fuel immune cell proliferation and phagocytosis in plasma.^26^ A significant reduction in amino acid levels after SARS-CoV-2 infection indicated strong immune activation in these patients.

### Combined biomarker signatures to predict COVID-19 disease severity in patients

**Fig. 4:**
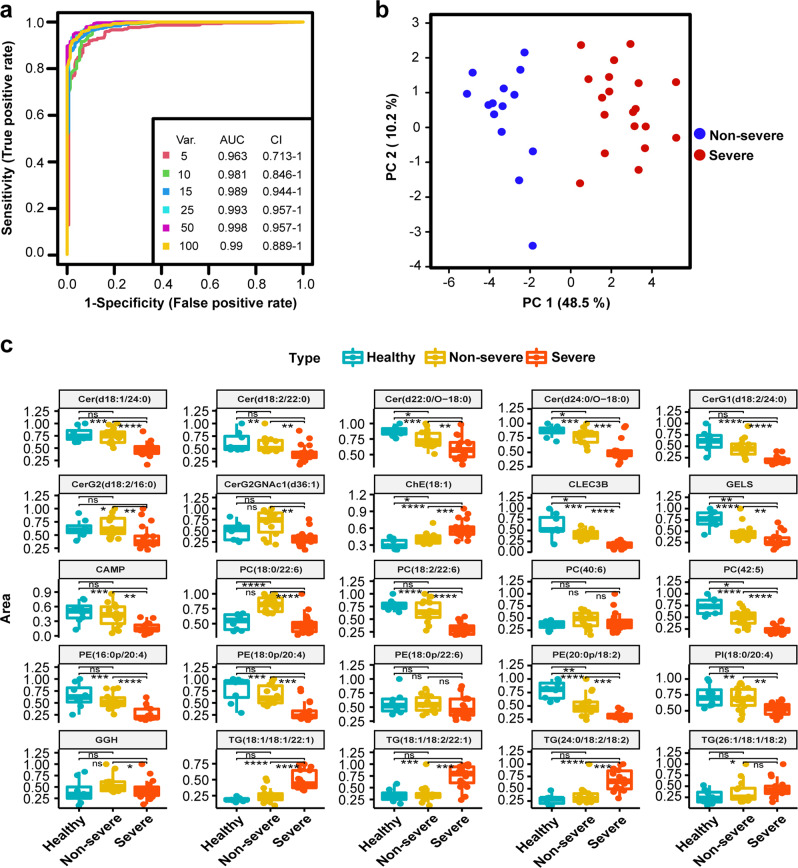
Biomarker analysis based on multi-omics signatures. **a** ROC curve analysis for the predictive power of combined multiple omics signatures selected by random forest for distinguishing non-severe from severe group. **b** Principle component analysis for the non-severe and severe groups based on selected 25 signatures. **c** Normalized selected signatures expression values for each sample from individual non-severe patients or severe COVID-19 patients

Proteome, amino acids, and lipidome levels were differentially regulated during SARS-CoV-2 infection. We further used these omics’ signatures to investigate the possibility for prognosis of disease severity. There were three cohorts for biomarker discovery and validation as described in Fig. 1a. For the training cohort, we built a random forest machine learning model based on proteome, amino acids, and lipidome data, 25 important variables including 4 proteins and 21 lipids were preferentially selected (Fig. 4a). This model reached an AUC of 0.993 with 95% CI at 0.957–1 in the training set (Fig. 4a) and all samples could be classified into right group (Fig. 4a). Principle component analysis based on these 25 molecule panels could divide samples into right groups, as shown in Fig. 4b. These molecular signatures for classification included four ceramides (Cer (d18:1/24:0), Cer (d18:2/22:0), Cer (d22:0/O-18:0), and Cer (d24:0/O-18:0)), three glycosylceramides (CerG1 (d18:2/24:0), CerG2 (d18:2/16:0), and CerG2GNAc1 (d36:1)), one cholesterol ester (ChE (18:1)), four phosphocholines (PC (18:0/22:6), PC (18:2/22:6), PC (40:6), and PC (42:5)), four phosphatidylethanolamines (PE (16:0p/20:4)), PE (18:0p/20:4), PE (18:0p/22:6), and PE (20:0p/18:2)), one phosphatidylinositol (PI (18:0/20:4)), four triacylglycerides (TG (18:1/18:1/22:1), TG (18:1/18:2/22:1), TG (24:0/18:2/18:2), and TG (26:1/18:1/18:2)), and four proteins (CLEC3B, GELS, CAMP, and GGH). Among these molecular signatures, CLEC3B is a lung tissue-enriched protein, and a potential diagnostic and prognostic biomarker in lung cancer and association with pulmonary immune microenvironment.^27^ The corresponding expression pattern of each molecule in healthy controls, non-severe, and severe patients was shown in Fig. 4c. There were 20 molecules downregulated in the severe group as compared with the non-severe cohorts, and five molecules upregulated in the severe group, including PC (18:2/22:6) and four TGs.

**Fig. 5:**
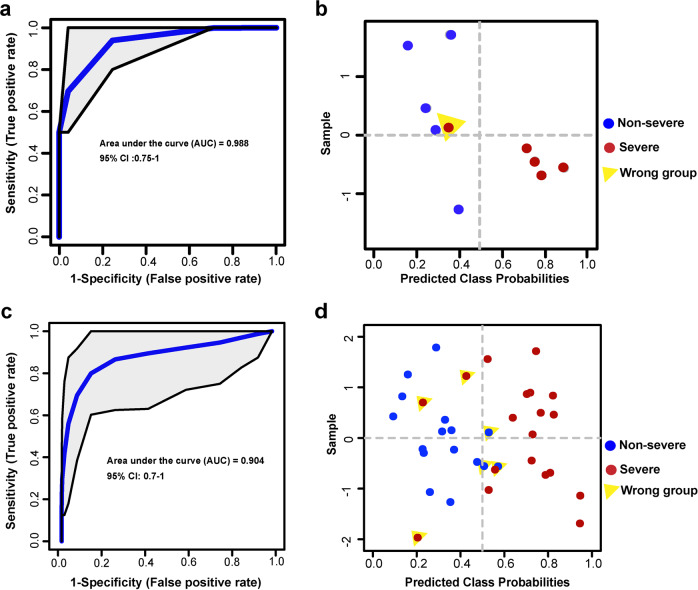
Validation performance in validation cohort 1 and validation cohort 2. **a** ROC curve analysis for the predictive power of validated lipid signatures in new plasma samples. **b** Performance of the model in new plasma cohort of ten COVID-19 patients. Samples classified into wrong group were labeled. **c** ROC curve analysis for the predictive power of validated lipid signatures in urine samples. **d** Performance of the model in urine cohort of ten COVID-19 patients. Samples classified into wrong group were labeled

To validate the prediction power of these 25 molecular signatures in plasma selected as potential indicators to distinguish non-severe and severe patients, their abundances in new plasma samples from an independent cohort of ten patients (containing five non-severe and five severe patients) were explored. The prediction was evaluated by the ROC analysis, as shown in Fig. 5a, the AUC could reach to 0.988 with 95% CI (0.75–1), using these 25 molecules. One patient was classified into the wrong group as highlighted in Fig. 5b. Clinical retrospective analysis showed that this patient only required noninvasive ventilation (other severe patients required invasive ventilation), and was not exacerbated after ICU admission.

In order to further evaluate the performance of the biomarker panels identified in this study, we further explored available public datasets. In this study, we adapted relative complemented techniques enriching more small proteins and preferring to more lipid classes compared with previous publications.^12,28^ We were able to identify several novel molecular signatures for COVID-19, which have never been reported before. Therefore, we only found one dataset which is suitable for validation.^29^ Using this dataset, we were able to quantify 19 molecules among the 25 molecular signatures, the AUC could reach to 0.901 (95% CI at 0.807–0.973) for classifying non-severe (*n* = 54) to severe group (*n* = 21; Supplemental Fig. 8). These results indicated that the performance appeared well accepted in the independent cohort study.

In addition to plasma validation, potential prediction power of these molecules in urine was also investigated, as urine samples were acquired noninvasively and also reflected dynamic changes of disease.^30^ At last, eight molecules were able to be quantified in urine samples, because some lipids were not secreted in urine, or the abundance was lower than technique detection threshold. The prediction was evaluated by the ROC analysis. As shown in Fig. 5c, the AUC could reach to 0.904 with 95% CI (0.7–1). Samples classified into the wrong group were highlighted in Fig. 5d. Three severe samples classified into the non-severe group were acquired from one patient with three serial samplings across the whole admission. Three non-severe samples classified into the severe group were acquired from two patients with two serial samplings across the whole admission. Overall, the precise prediction in urine proved the feasibility of dynamic monitoring of patients.

### A working model of multi-omics changes in SARS-CoV-2 infection

We summarized the differential expression factors in the plasma of COVID-19 patients, as illustrated in Supplementary Fig. 9a. Critical proteins in viral infection and inflammatory response, include host defense peptides like DEFA1, apoliproteins (APOs), and interferon-stimulated genes like SAA1 as uncharacterized antiviral gene,^31^ accurate phase proteins like complement C6, and the corresponding lipids or amino acids also play functions in the regulation, such as PCs, TGs, PS, and amino acids, as shown in Supplementary Fig. 9b. APOs, such as Apo E, acts as inducible inhibitor of viral production and infectivity in macrophages. The decreased abundance of PCs in plasma of COVID-19 patients revealed the aberration of macrophage choline cytidylyltransferase α, which used PC to generate CDP-choline.^32^ The interaction between PS-expressing cells and immune cells triggers immunosuppressive pathways,^33^ so the upregulated PSs in plasma revealed the presence of immunosuppression during infection. We further speculated the possible pathogenesis of COVID-19 in the following discussion section.

### Potential therapeutic drug target discovery against SARS-CoV-2 infection

Proteome, amino acids, and lipidome profiling could also provide potential therapeutic drug targets, because the abundance change of molecular signatures in plasma reflected the virus–host interaction during SARS-CoV-2 infection. Herein, we analyzed potential drug targets based on proteins enriched in lung tissue and targets of Food and Drug Administration (FDA)-approved drugs as annotated in HPA.^14^ We identified five lung-enhanced proteins, including MRC1, histone H4, CCL18, SG3A1, and CLEC3B, annotated as “drug targets” in Fig. 2b. Interestingly, MRC1 (CD206) is a key C-type lectin receptor expressed on the surface of M2 macrophages and used as a surface marker for M2 macrophages. MRC1 also has an anti-inflammatory function and induces immunotolerance.^34^ CCL18 (AMAC1, MIP4) is a chemokine that attracts lymphocytes, including CD4^+^ and CD8^+^ T cells, which might play a role in both humoral andcellular immune responses.^35^ A previous study showed that all COVID-19 patients with severe respiratory failure displayed either immune dysregulation or macrophage activation syndrome.^36^ Modulating macrophage activation might be a potential therapeutic strategy for severe COVID-19 patients. MRC1, the target of metformin which has been approved to suppress M2-like polarization of macrophages, was also proposed as potential drug candidate for COVID-19 treatment.

In addition to MRC1, other targets of FDA-approved drugs were also identified, including S100A8, S100A9, FGB, C5, ITGB3, KLKB1 (plasma kallikrein; PK), and ANXA1. As blockade of S100A8/S100A9 reduces pro-inflammatory cytokine production and ameliorates excessive inflammation responses.^37^ Resveratrol, one of FDA-approved drugs targeting ITGB3, was reported to be a combination medicine for COVID-19 treatment.^38^ In addition, resveratrol also has antiviral effects for MERS-CoV.^39^ Inhibiting PK might prevent acute respiratory distress syndrome of COVID-19 patients.^40^ ANXA1 targeted drug dexamethasone, hydrocortisone, and prednisolone have been used to treat severely ill COVID-19 patients.^41^ FGB targeted drug, dipyridamole, which has anticoagulant effect and also had been proved to be a potential therapeutic drug for COVID-19 patients.

**Fig. 6:**
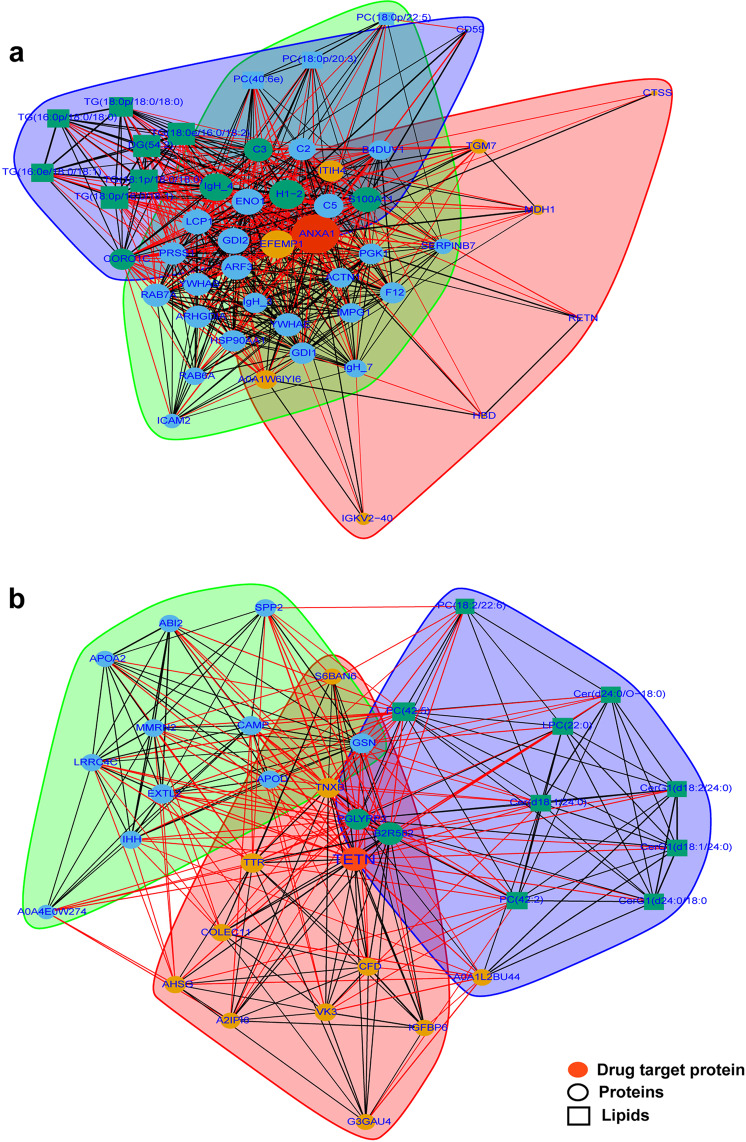
Drug target analysis by interaction among molecules. **a** Interaction between target protein-ANXA1 and other molecules include proteins, lipids, and amino acids. **b** Interaction between target protein-CLEC3B and other molecules include proteins, lipids, and amino acids

For these 12 proteins identified as therapeutic drug targets, we further explored their network relationships with other molecular signatures in plasma, including proteins, lipids, and amino acids. The basis for the network-based drug repurposing methodologies rests on that the key proteins localized in the corresponding subnetwork could interact with significantly dysregulated molecules and used as drug targets.^42^ As illustrated in Fig. 6a, for ANXA1, it could interact with complement molecules (C2, C3, and C5) and lipids (TGs), which played important roles in SARS-CoV-2 infection, especially in severe patients. In addition to ANXA1, which has been used to treat severely ill COVID-19 patients, another novel drug target like CLEC3B was also identified. As shown in Fig. 6b, CLEC3B could interact with host defense proteins (CAMP), Apo families (Apo A2, and Apo D), and lipids, such as CerG1 classes. CLEC3B could bind HMGB1 (the high-mobility group box-1) and reciprocally enhance macrophage endocytosis, thereby induce macrophage pyroptosis, which was proposed as a drug target for sepsis.^42^ As our data revealed, it could also be a potential drug target for COVID-19 therapy.

## Discussion

COVID-19 has led to the global pandemic, and represents a major threat to public health and global economy. So far, there is no effective treatment strategy to prevent death of severe patients. The diagnosis and prognosis of COVID-19 are important for proper healthcare resource allocation and selective treatment of severe patients.

Here, we comprehensively profiled molecular changes in plasma and urine of COVID-19 patients using quantitative proteome, amino acids, and lipids based on LC–MS platform, and we found a series of biomarkers. Random forest algorithm was applied to select representative molecular signatures, using proteins and lipids quantified from a training cohort with ten non-severe and ten severe patients. The model combined with 25 molecules, including 21 lipids and 4 proteins, could accurately classify severe patients with AUC reached to 0.993, and was further validated using 10 new plasma samples acquired from independent patients, 9 of which were correctly classified. In addition to plasma, these molecules were further validated in patients’ urine for diagnosis and prognosis. The accuracy was lower than plasma, as some molecule abundances were inconsistent with corresponding plasma. In addition to previous reported molecules detected by proteomics and metabolomics,^12^ more lipid signatures were identified. Detection of multiple proteins in plasma by mass spectrometry faces some difficulties, including relatively complicated sample preparation and interference from high-abundance proteins. The quantification of lipids in plasma could be suitable for the diagnosis and prognosis of the COVID-19 patients due to its simple sample preparation and detection procedures. In addition, we performed our proteome preparation by C18 enrichment method which could collect more small molecular proteins, and provided another insight for exploring infection-related small proteins, such as lung-enriched protein, CLEC3B.

In this study, there were no significant age difference between health donors (mean of ages is 42.0) and non-severe (mean of ages is 43.3) patients, while the severe patients were older (mean of ages is 62.1). To determine the effect of age on the results, we analyzed the 25 biomarkers in different groups (health donors, non-severe, and severe patients), using two-way ANOVA to explore the two-factor impact on these biomarkers. The results showed that these signatures were mainly effected by COVID-19 infection as the *P* value for ages was not significant (*P* value >0.05; Supplementary Dataset 1).

After the virus invades lung tissue, innate immune response is activated and inflammatory cytokines are produced, such as IL-6 and TNF. Liver functions changes and further produces SAA1 and CRP, which have been identified as biomarkers for the severity in COVID-19 patients.^12^ SAA1 and CRP further activate the complement system to recruit inflammatory cells, increase vascular permeability, and contributing to cytokine storm. On the other hand, hyper-inflammatory response leads to lung damage, then activates M2 macrophage to promote fibers and collagen production and tissue remodeling.^43^ As showed in Supplementary Fig. 9, excessive activation of M2 macrophages consumed more glutamine and amino acid, and resulted in decreased concentration of glutamine in plasma. CCL18 served as a marker for enhanced risk of pulmonary fibrosis development.^44^ In addition, LL37, the unique cathelicidin in humans, was also downregulated in severe patients. LL37 was produced by epithelial tissues, as well as the innate immune system, such as human neutrophils, monocytes/macrophages, lymphocytes, mast cells, etc.,^45^ and it was identified as a broad-spectrum antimicrobial factor and used to inhibit respiratory syncytial virus infection.^46^

S100A8 and S100A9 are Ca^2+^-binding proteins that generally expressed in neutrophils and monocytes as a Ca^2+^ sensor in form of heterodimer and were upregulated in severe patients. S100A8/S100A9 can induce the secretion of multiple inflammatory cytokines, such as TNF, IL-6, and IL-1 via different signaling pathways.^37^ As blockade of IL-6 receptor by tocilizumab was reported as a therapeutic strategy for COVID-19 patients, inhibition of IL-6 secretion by blocking S100A8/S100A9 provides alternative treatment. Kallikrein/kinin system consists of two different proteolytic pathways: PK pathway and tissue kallikrein pathway. PK is primarily produced in liver, and complexed with high molecular weight kininogen in the plasma. PK is activated within the vasculature and releases bradykinin, the latter binds to bradykinin B2 receptors to enhance vascular permeability, triggering pain and pulmonary edema.^47^ Therefore, inhibition of PK might be a potential strategy to prevent acute respiratory distress syndrome.^40^

In summary, this study combined analysis of proteins, amino acids, and lipids in plasma and urine from COVID-19 patients, and revealed the molecular signatures related with SARS-CoV-2 infection, especially for the dysregulation of macrophage, proteins, and metabolites. A panel combined with 25 molecules proposed feasibly biomarkers for the prediction of the non-severe and severe COVID-19 patients. Several therapeutic drug targets were also identified according to the molecular signatures in plasma, such as metformin, resveratrol dexamethasone, and dipyridamole, which had potential to treat severely ill COVID-19 patients.

## Materials and methods

### Proteome analysis

Plasma samples were inactivated using UV for 1 h followed by processing with SPE columns (Agela, China) following to the manufacturer’s instructions, which removes high-abundance proteins and enriched low-abundance small protein with some modifications.^48^ Protein concentration was determined by the Bradford protein assay kit (Bio-Rad, USA), and subsequently reduced by dithiothreitol at 37 °C water bath for 30 min and alkylated by iodoacetamide at room temperature for 30 min in the darkroom. Proteins were digested by trypsin (Promega, USA) following FASP (filter-aided sample preparation) protocol.^49^ Samples were quantified using DIA mode by QExactive HF-X mass spectrometer (Thermo Scientific, San Jose, USA) coupled with an Ultimate 3000 UHPLC liquid chromatography (Thermo Scientific, San Jose, USA). Peptides were separated by self-packed analytical column (150 μm internal diameter, 1.7 μm particle size, and 35 cm column length) at the flow rate of 500 nL/min. The mobile phase A consists 0.1% formic acid in water; and the mobile phase B consists 0.1% formic acid in acetonitrile with 120 min elution gradient following settings as: 0–5 min, 5% B; 5–95 min, 5–25% B; 95–105 min, 25–35% B. For HF-X settings, the mass range of MS1 was set as 400–1250 *m*/*z* at the resolution of 120,000 with 50 ms max injection time. For the DIA setting, mass range of 400–1250 *m*/*z* was equally divided into 45 continuous windows MS2 scans at 30,000 resolution with the automatic max injection time and automatic gain control (AGC) of 1E6. Normalized collision energy of MS2 was distributed to 22.5, 25, and 27.5.

The raw data were analyzed by Spectronaut software (v12.0, Biognosys, Switzerland) with the default settings against the self-built plasma spectral library. The FDR cutoff was set as 1% at both peptide and protein levels. For differential analyses, the R package MSstats^50^ was used for log2 transformation, normalization, and *P* value calculation.

### Lipidome and amino acids analysis in plasma

Lipids extraction was primarily performed according to previously reported methods.^51^ In short, 100 µL plasma samples were extracted by directly adding 300 µL of precooled isopropanol, and internal standards (SPLASH® LIPIDOMIX® Mass Spec Standard, Avanti, USA) were added for the quality control (QC) of sample preparation. After vortex for 1 min and incubate at −20 °C for overnight, samples were centrifuged for 20 min at 14,000 r.p.m., and the supernatants were transferred to autosampler vials for LC–MS analysis. A QC sample was prepared by pooling the same volume of each sample to evaluate the reproducibility of the whole LC–MS analysis.

The samples were analyzed on a Waters 2D UPLC (Waters, USA), coupled to a QExactive mass spectrometer (Thermo Fisher Scientific, USA) with a heated electrospray ionization source and controlled by the Xcalibur 2.3 software program (Thermo Fisher Scientific, Waltham, MA, USA). Chromatographic separation was performed on a Waters ACQUITY UPLC CSH C18 column (1.7 μm, 2.1 mm × 100 mm, Waters, USA), and the column temperature was maintained at 55 °C. The mobile phase consisted of acetonitrile/water (60:40, v/v), mixed with 10 mM ammonium formate and 0.1% formic acid (A) and isopropanol/acetonitrile (90:10, v/v), mixed with 10 mM ammonium formate and 0.1% formic acid (B) in the positive mode, and in the negative mode, acetonitrile/water (60:40, v/v), mixed with 10 mM ammonium formate (A) and isopropanol/acetonitrile (90:10, v/v), mixed with 10 mM ammonium formate (B). The gradient conditions were as follows: 0–5 min, 40–43% B; 5–5.1 min, 43–50% B; 5.1–18 min, 50–54% B; 18–18.1 min, 54–70% B; 18.1–27 min, 70–99% B; 27–27.1 min, 99–40% B; and 27.1–30 min, 40% B. The flow rate was 0.4 mL/min and the injection volume was 5 μL.

The mass spectrometric settings for positive/negative ionization modes were as follows: spray voltage, 3.8/–3.2 kV; sheath gas flow rate, 40 arbitrary units (arb); aux gas flow rate, 10 arb; aux gas heater temperature, 350 °C; capillary temperature, 320 °C. The full scan range was 200–2000 *m*/*z* with a resolution of 70,000, and the AGC target for MS acquisitions was set to 3e6 with a maximum ion injection time of 100 ms. Top three precursors were selected for subsequent MSMS fragmentation with a maximum ion injection time of 50 ms and resolution of 17,500, the AGC was 1e5. The stepped normalized collision energy was set to 15, 30, and 45 eV. LipidSearch 4.1 SP2 software (Thermo Fisher, USA) was used for lipid identification and quantitation. The quantified result was further processed using metaX package.

### Lipids validated in urine by MRM quantification

For semiquantitative assay of the 21 potential lipid markers in the urine of COVID-19 patients, lipids in urine were extracted using precooled isopropanol similar with plasma, as described above. The 21 lipid markers were quantified with multiple reaction monitoring (MRM) mode by QTRAP 5500 (SCIEX, USA) coupled with UPLC (Waters, USA) using same LC condition, as described in discovery stage. Data were processed using MultiQuant software (SCIEX, USA).

### Combined biomarkers validated in new plasma cohort by PRM quantification

Plasma samples were prepared for protein and lipid extraction following the above description, and analyzed by targeted quantification, parallel reaction monitoring (PRM). For protein quantification, PRM was acquired on the same UPLC–MS system (Ultimate 3000 UPLC coupled with QE HF-X) with the previous proteome profiling. All PRM data were processed using skyline (v20.1).

### Statistical analysis

The time series analysis for proteins and lipids were applied based on fuzzy *c*-means algorithm implemented in the R package (version 2.48.0) Mfuzz. Optimized number of clusters was estimated by calculating minimum centroid distance. Metaboanalyst was used for biomarker analysis using the multivariate ROC curve analyses based on random forests algorithms. All figures were drawn using corresponding R packages.

## Supplementary information

Supplementary Materials

Supplementary Dataset 1

Supplementary Dataset 2

## Data Availability

All data needed to evaluate the conclusions in the paper are present in the paper and/or the Supplementary Materials. Additional data related to this paper may be requested from the authors.

## References

[1] W.H.O. Coronavirus disease (COVID-19) dashboard. https://covid19.who.int/ (2020).

[2] Richardson S (2020). Presenting characteristics, comorbidities, and outcomes among 5700 patients hospitalized with COVID-19 in the New York City Area. JAMA.

[3] Livingston E, Bucher K (2020). Coronavirus disease 2019 (COVID-19) in Italy. JAMA.

[4] Wu Z, McGoogan JM (2020). Characteristics of and important lessons from the Coronavirus disease 2019 (COVID-19) outbreak in China: summary of a report of 72314 cases from the Chinese Center for Disease Control and Prevention. JAMA.

[5] Chi, Y. et al. Serum cytokine and chemokine profile in relation to the severity of Coronavirus disease 2019 (COVID-19) in China. *J. Infect. Dis.* **222**, 746–754 (2020). 10.1093/infdis/jiaa363 PMC7337752 32563194

[6] Huang, W. et al. Lymphocyte subset counts in COVID-19 patients: a meta-analysis. *Cytometry A*. **97**, 772–776 (2020). 10.1002/cyto.a.24172 PMC7323417 32542842

[7] Xiong Y (2020). Transcriptomic characteristics of bronchoalveolar lavage fluid and peripheral blood mononuclear cells in COVID-19 patients. Emerg. Microbes Infect..

[8] Buja LM (2020). The emerging spectrum of cardiopulmonary pathology of the coronavirus disease 2019 (COVID-19): Report of 3 autopsies from Houston, Texas, and review of autopsy findings from other United States cities. Cardiovasc. Pathol..

[9] Vijayvargiya, P. et al. Treatment considerations for COVID-19: a critical review of the evidence (or Lack Thereof). *Mayo Clin. Proc.* **95**, 1454–1466 (2020). 10.1016/j.mayocp.2020.04.027 PMC7190528 32561148

[10] Matthay MA, Aldrich JM, Gotts JE (2020). Treatment for severe acute respiratory distress syndrome from COVID-19. Lancet Respir. Med..

[11] Amemiya T, Gromiha MM, Horimoto K, Fukui K (2019). Drug repositioning for dengue haemorrhagic fever by integrating multiple omics analyses. Sci. Rep..

[12] Shen B (2020). Proteomic and metabolomic characterization of COVID-19 patient sera. Cell.

[13] Lorizate M, Kräusslich HG (2011). Role of lipids in virus replication. Cold Spring Harb. Perspect. Biol..

[14] Uhlén M (2015). Proteomics. Tissue-based map of the human proteome. Science.

[15] Uhlen, M. et al. A genome-wide transcriptomic analysis of protein-coding genes in human blood cells. *Science* **366**, eaax9198 (2019). 10.1126/science.aax9198 31857451

[16] UniProt Consortium. UniProt: a worldwide hub of protein knowledge. *Nucleic Acids Res*. **47**, D506–D515, (2019). 10.1093/nar/gky1049 PMC6323992 30395287

[17] Sproston NR, Ashworth JJ (2018). Role of C-reactive protein at sites of inflammation and infection. Front. Immunol..

[18] Masson D, Jiang XC, Lagrost L, Tall AR (2009). The role of plasma lipid transfer proteins in lipoprotein metabolism and atherogenesis. J. Lipid Res..

[19] Khovidhunkit W, Memon RA, Feingold KR, Grunfeld C (2000). Infection and inflammation-induced proatherogenic changes of lipoproteins. J. Infect. Dis..

[20] Eisfeld AJ (2017). Multi-platform ‘omics analysis of human ebola virus disease pathogenesis. Cell Host Microbe.

[21] Nomura S, Shimizu M (2015). Clinical significance of procoagulant microparticles. J. Intensive Care..

[22] Deguchi H (2017). Low level of the plasma sphingolipid, glucosylceramide, is associated with thrombotic diseases. Res. Pract. Thromb. Haemost..

[23] von Gerichten J (2017). Diastereomer-specific quantification of bioactive hexosylceramides from bacteria and mammals. J. Lipid Res..

[24] Zuo, T. et al. Alterations in fecal fungal microbiome of patients with COVID-19 during time of hospitalization until discharge. *Gastroenterology.* **159**, 1302–1310 (2020). 10.1053/j.gastro.2020.06.048 PMC7318920 32598884

[25] Wannemacher RW (1977). Key role of various individual amino acids in host response to infection. Am. J. Clin. Nutr..

[26] Cruzat, V. et al. Glutamine: metabolism and immune function, supplementation and clinical translation. *Nutrients*. **10**, 1564 (2018). 10.3390/nu10111564 PMC6266414 30360490

[27] Sun J (2020). CLEC3B as a potential diagnostic and prognostic biomarker in lung cancer and association with the immune microenvironment. Cancer Cell Int..

[28] Song JW (2020). Omics-driven systems interrogation of metabolic dysregulation in COVID-19 pathogenesis. Cell Metab..

[29] Peng Wu, D. C. et al. The Trans-omics Landscape of COVID-19. Preprint at *medRxiv*10.1101/2020.07.17.20155150 (2020).

[30] Gao Y (2013). Urine-an untapped goldmine for biomarker discovery?. Sci. China Life Sci..

[31] Quicke KM, Suthar MS (2013). The innate immune playbook for restricting West Nile virus infection. Viruses.

[32] Sanchez-Lopez E (2019). Choline uptake and metabolism modulate macrophage IL-1β and IL-18 production. Cell Metab..

[33] Birge RB (2016). Phosphatidylserine is a global immunosuppressive signal in efferocytosis, infectious disease, and cancer. Cell Death Differ..

[34] Azad, A. K., Rajaram, M. V. & Schlesinger, L. S. Exploitation of the macrophage mannose receptor (CD206) in infectious disease diagnostics and therapeutics. *J. Cytol. Mol. Biol*. **1**, 1000003 (2014). 10.13188/2325-4653.1000003 PMC3963702 24672807

[35] Luzina IG (2006). Induction of prolonged infiltration of T lymphocytes and transient T lymphocyte-dependent collagen deposition in mouse lungs following adenoviral gene transfer of CCL18. Arthritis Rheum..

[36] Giamarellos-Bourboulis EJ (2020). Complex Immune Dysregulation in COVID-19 Patients with Severe Respiratory Failure. Cell Host Microbe.

[37] Wang S (2018). S100A8/A9 in inflammation. Front. Immunol..

[38] Marinella, M. A. Indomethacin and resveratrol as potential treatment adjuncts for SARS-CoV-2/COVID-19. *Int. J. Clin. Pract.* **74**, e13535 (2020). 10.1111/ijcp.13535 PMC7261995 32412158

[39] Lin SC (2017). Effective inhibition of MERS-CoV infection by resveratrol. BMC Infect. Dis..

[40] van de Veerdonk, F. L. et al. Kallikrein-kinin blockade in patients with COVID-19 to prevent acute respiratory distress syndrome. *eLife* **9**, e57555 (2020). 10.7554/eLife.57555 PMC7213974 32338605

[41] Ledford, H. Coronavirus breakthrough: dexamethasone is first drug shown to save lives. *Nature* **582**, 469 (2020). 10.1038/d41586-020-01824-5 32546811

[42] Zhou Y (2020). Network-based drug repurposing for novel coronavirus 2019-nCoV/SARS-CoV-2. Cell Discov..

[43] Laskin DL, Malaviya R, Laskin JD (2019). Role of macrophages in acute lung injury and chronic fibrosis induced by pulmonary toxicants. Toxicol. Sci..

[44] Prasse A (2007). CCL18 as an indicator of pulmonary fibrotic activity in idiopathic interstitial pneumonias and systemic sclerosis. Arthritis Rheum..

[45] Bandurska K, Berdowska A, Barczyńska-Felusiak R, Krupa P (2015). Unique features of human cathelicidin LL-37. BioFactors.

[46] Currie SM (2013). The human cathelicidin LL-37 has antiviral activity against respiratory syncytial virus. PLoS ONE.

[47] Masuda T, Shimazawa M, Hara H (2015). The kallikrein system in retinal damage/protection. Eur. J. Pharmacol..

[48] Josić D (1998). Size-exclusion chromatography of plasma proteins with high molecular masses. J. Chromatogr. A.

[49] Wiśniewski JR, Zougman A, Nagaraj N, Mann M (2009). Universal sample preparation method for proteome analysis. Nat. Methods.

[50] Choi M (2014). MSstats: an R package for statistical analysis of quantitative mass spectrometry-based proteomic experiments. Bioinformatics.

[51] Zeng C (2017). Lipidomics profiling reveals the role of glycerophospholipid metabolism in psoriasis. GigaScience.

