# Supplementary material for: Multi-platform omics analysis reveals molecular signature for COVID-19 pathogenesis, prognosis and drug target discovery
Source: Signal Transduct Target Ther. 2021 Apr 15;6:155. doi: 10.1038/s41392-021-00508-4 (PMC8047575; doi:10.1038/s41392-021-00508-4)
Supplement: Supplementary file 1 — Supplementary Materials [file 41392_2021_508_MOESM1_ESM.docx]

Supplementary Materials for

Multi-Platform Omics Analysis Reveals Molecular Signature for COVID-19 Pathogenesis, Prognosis and Drug Target Discovery

Yuming Li^1^*, Guixue Hou^2^*, Haibo Zhou^3^*, Yanqun Wang^1^*, Airu Zhu^1^*, Fei Xiao^4^*, Shanwen Lin^5^*, Dongdong Liu^1^*, Dunrong Zhou^5^, Lang Mai^6^, Lu Zhang^7,8^, Zhaoyong Zhang^1^ Lijun Kuang^1^, Jiao Guan^2^, Qiushi Chen^2^, Liyan Wen^1^, Yanjun Zhang^1^, Jianfen Zhuo^1^, Fang Li^1^, Zhen Zhuang^1^, Zhao Chen^1^, Ling Luo^1^, Donglan Liu^1^, Chunke Chen^1^, Mian Gan^1^, Nanshan Zhong^1^, Hein Min Tun^9†^, Jingxian Zhao^1†^, Jincun Zhao^1,7†^, Yan Ren^2†^, Yonghao Xu^1†^

Correspondence to: [dryonghao@163.com](mailto:dryonghao@163.com); [reny@genomics.cn](mailto:reny@genomics.cn); [zhaojincun@gird.cn](mailto:zhaojincun@gird.cn); [zhaojingxian@gird.cn](mailto:zhaojingxian@gird.cn); heinmtun@hku.hk

**This PDF file includes:**

Tables. S1

Figures. S1 to S9

**Other Supplementary Materials for this manuscript include the following:**

Dataset S1 to S2

Supplementary Dataset 1, available online only;

Supplementary Dataset 2, available online only.

# Table S1. (separate file)

# Figure. S1.

**
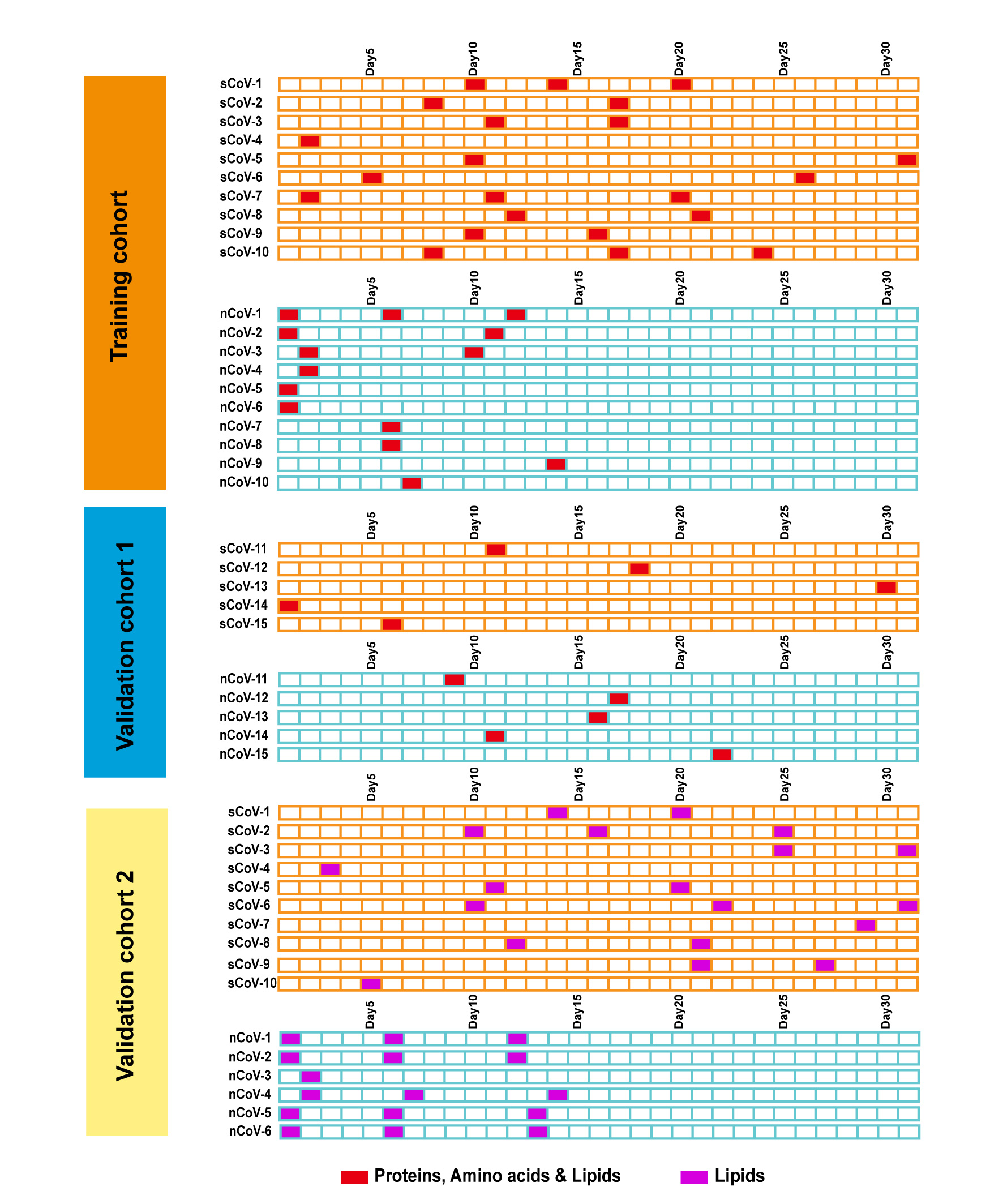
**

**Fig. S1. Sampling information for COVID-19 patients in the training cohort, validation cohort 1 and cohort 2.** s-nCoV represents severe patients infected with SARS-CoV-2, and n-nCoV represents non-severe patients infected with SARS-CoV-2.

# Figure. S2.


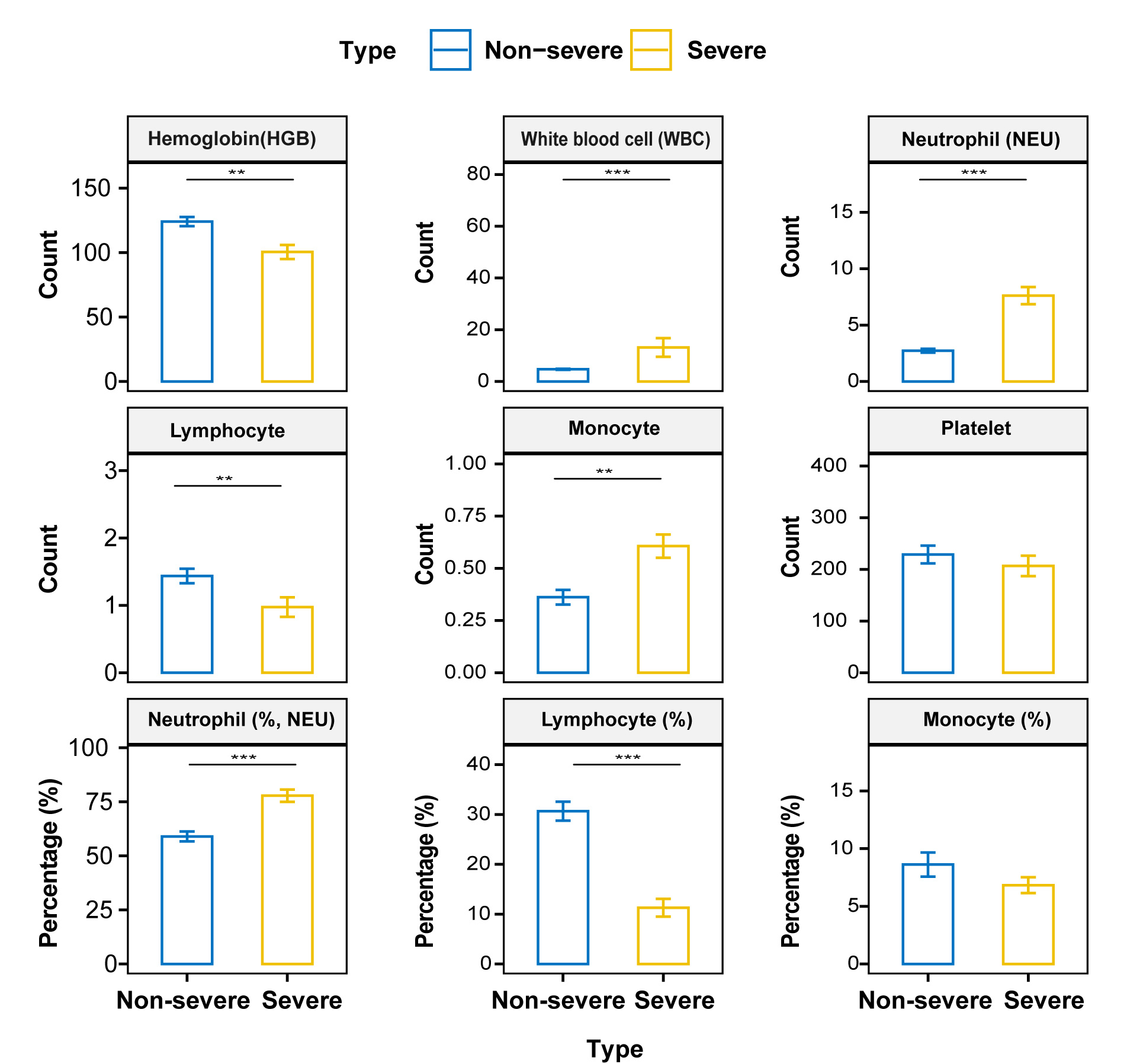


**Fig. S2. Clinical information for COVID-19 patients (according to training dataset)**.

# Figure. S3.

**
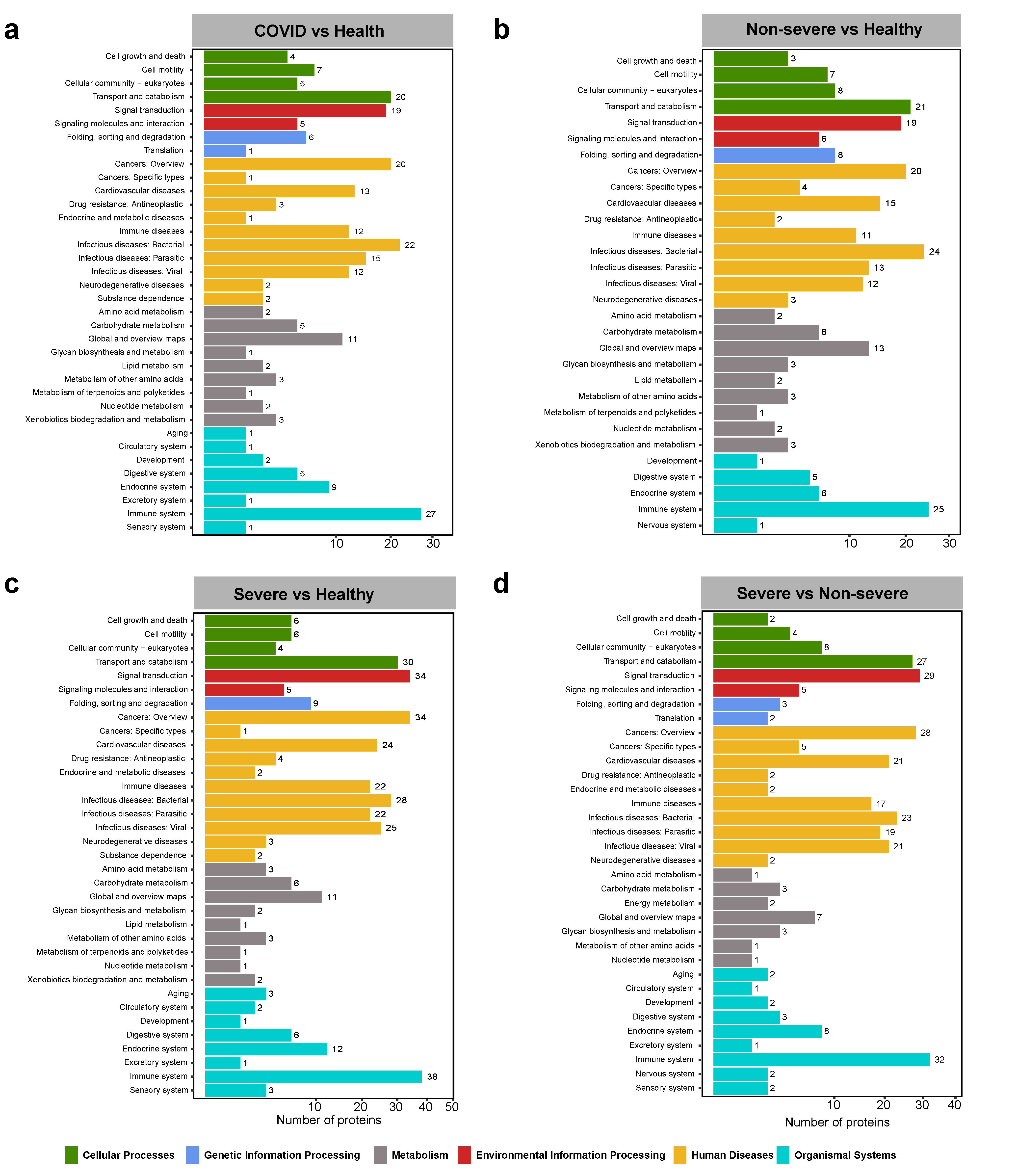
**

**Fig. S3. Pathways enriched for differential proteins.** (**A**) Pathways enriched for differential proteins between COVID-19 and Healthy group. (**B**) Pathways enriched for differential proteins between Non-severe and Healthy group. (**C**) Pathways enriched for differential proteins between Severe and Healthy group. (**D**) Pathways enriched for differential proteins between Severe and Non-severe group.

# Figure. S4.

**
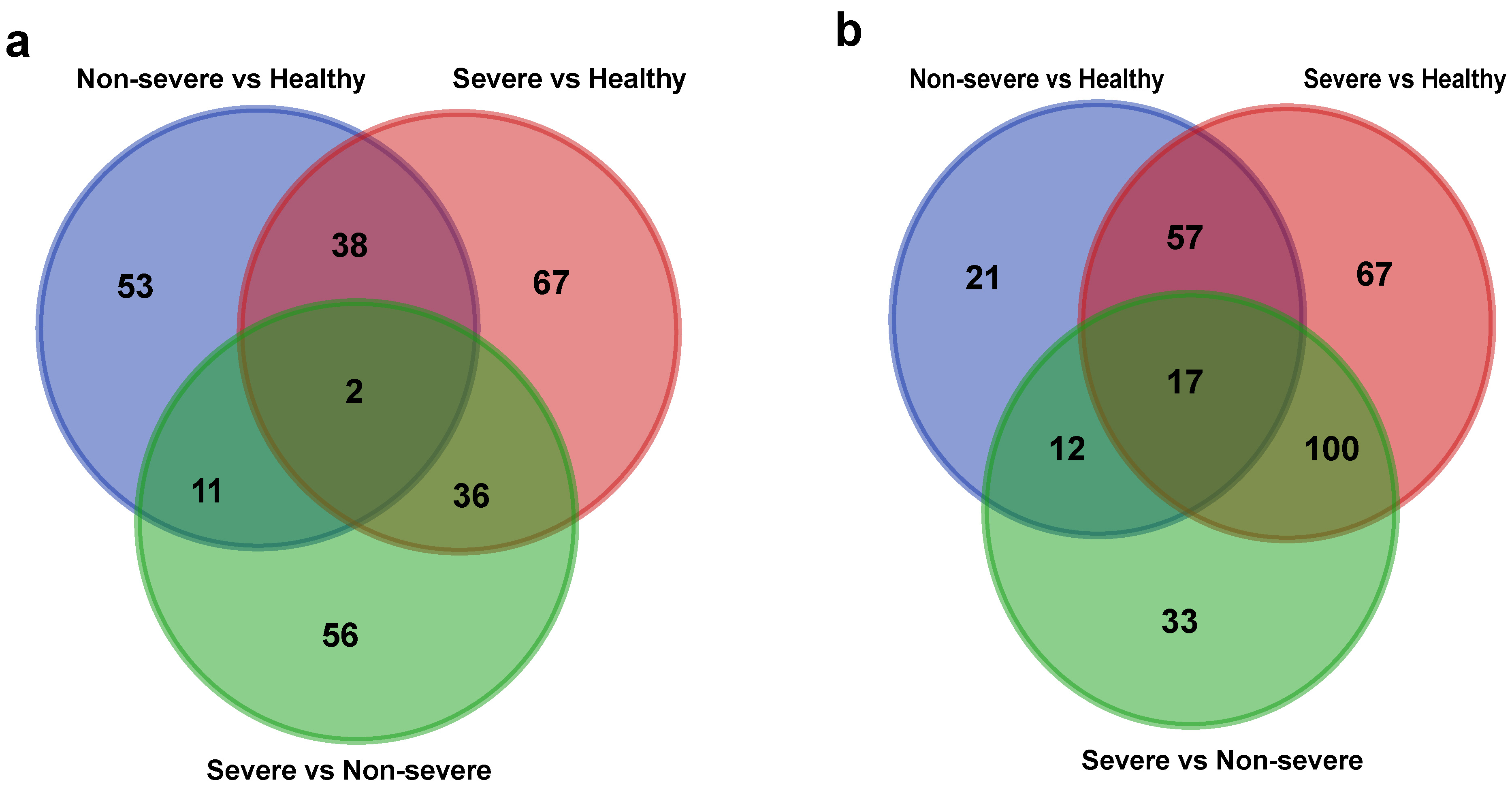
**

**Fig. S4. Overlap of differential proteins and lipids between three comparison.** (**A**) Overlap of differential proteins between three comparison. (**B**) Overlap of differential proteins between three comparison.

# Figure. S5.


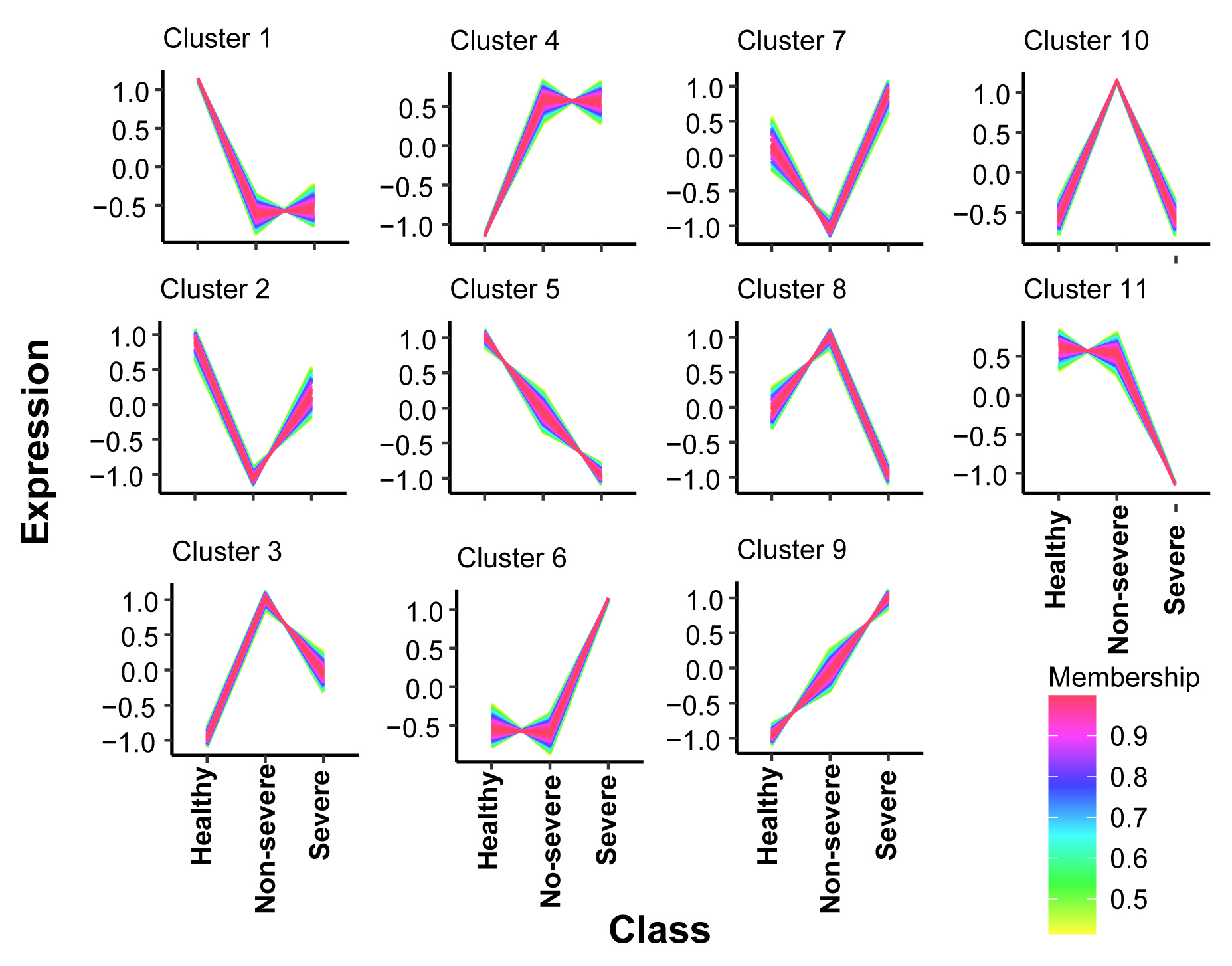


**Fig. S5. Time clustering analysis of all quantified proteins from healthy, non-severe to severe groups.**

# Figure. S6.


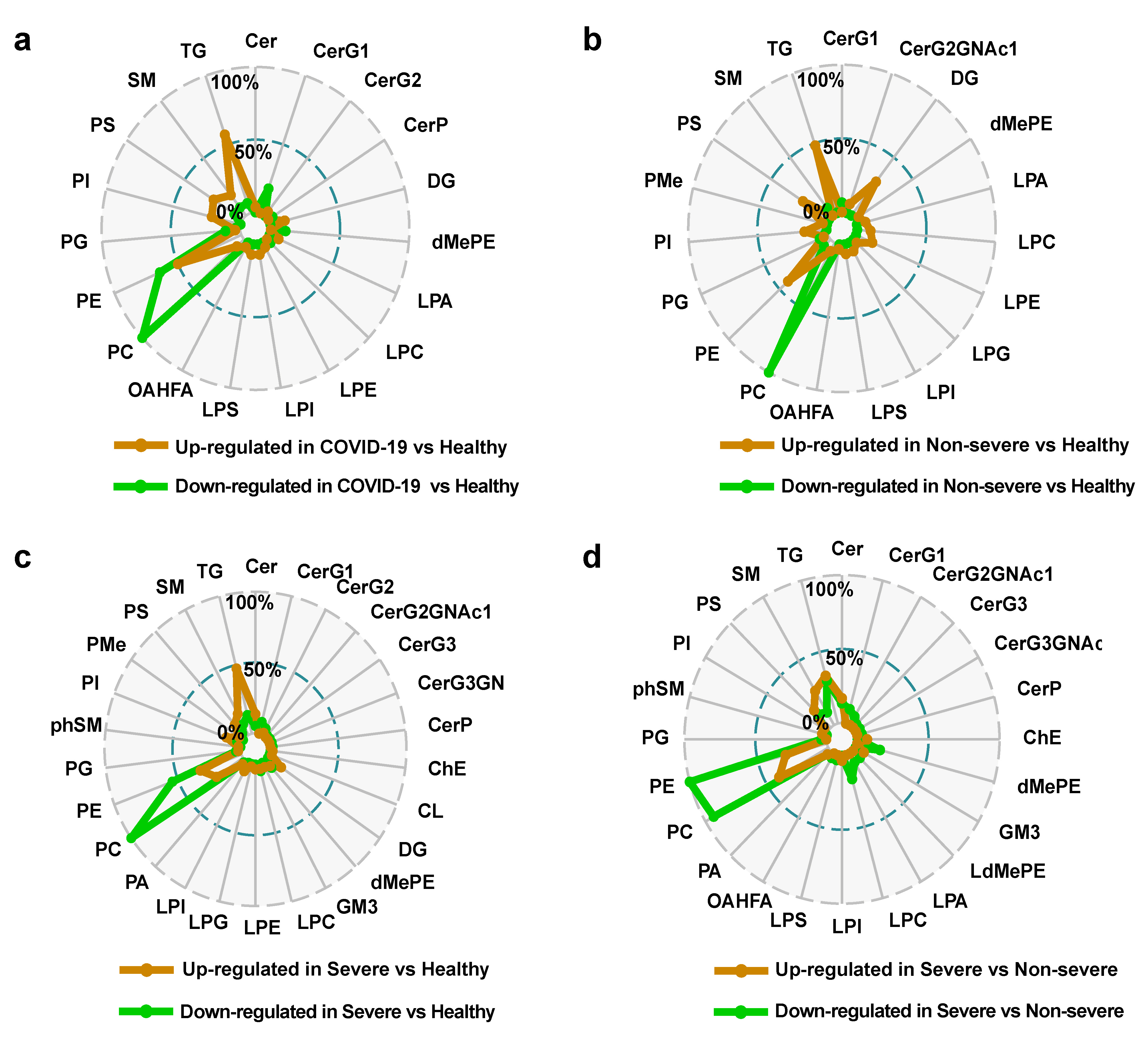


**Fig. S6. Different lipid classes related with COVID-19.** (**A**) The number of significantly changed (Fold changes >1.5, P < 0.01) plasma lipid species were increased (red line) or decreased (blue line) in COVID-19 patients in at least one comparison (Non-severe vs. Healthy, Severe vs. Health) for different lipid subclasses. The total number of significantly changed lipid species for each lipid subclass are shown by the dark gray line and are indicated in parentheses below each label at the edge of the radar map. The highest value depicted by the radar map is 30 (outermost concentric circle in light grey), and the line corresponding to 20 is labeled on the panel. (**B**) The proportion of lipid species for each lipid subclass that exhibit significantly higher (red line) or lower (blue line) expression in Non-severe fatalities relative to Healthy (Fold changes >1.5, P < 0.01). The highest value shown by the radar map is 100% (outermost concentric circle in light grey), and the line corresponding to 50% is labeled on the panel. CE, cholesterol ester; Cer, ceramide; HexCer, monohexosylceramide; GM3, ganglioside GM3; SM, sphingomyelin; DG, diacylglycerides; MG, monoacylglycerolipids; TG, triacylglycerides; PC, phosphocholine; LPC, lysophosphocholine; PE, phosphoethanolamine; LPE, lysophosphoethanolamine; PG, phosphoglycerol; PI, phosphatidylinositols; PS, phosphoserine. (**C**) The proportion of lipid species for each lipid subclass that exhibit significantly higher (red line) or lower (blue line) expression in Severe fatalities relative to Healthy (Fold changes >1.5, P < 0.01). (**D**) The proportion of lipid species for each lipid subclass that exhibit significantly higher (red line) or lower (blue line) expression in Severe fatalities relative to Non-severe (Fold changes >1.5, P < 0.01).

# Figure. S7.


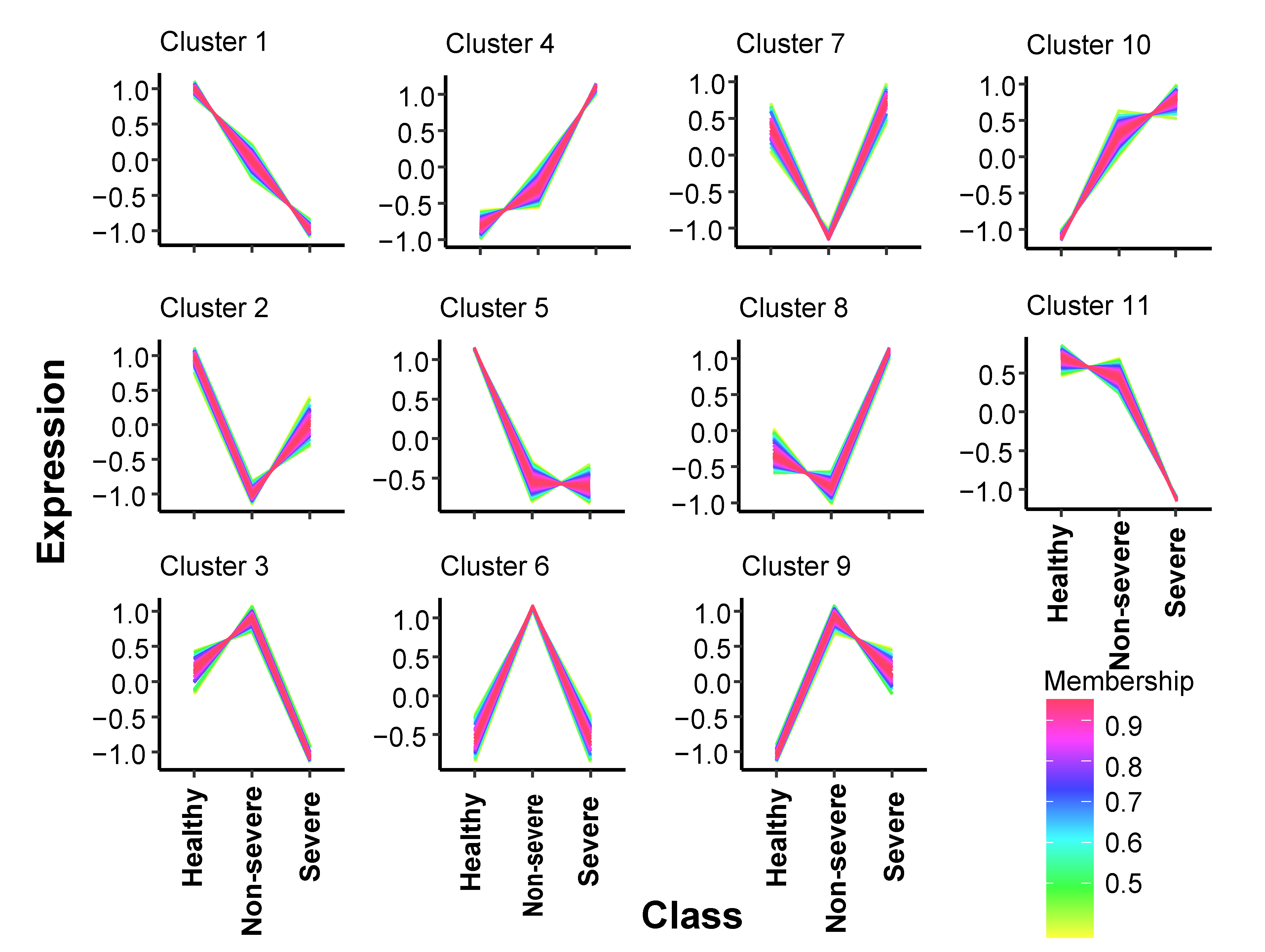


**Fig. S7. Time clustering analysis of all quantified lipids from healthy, non-severe to severe groups.**

**Figure. S8.**


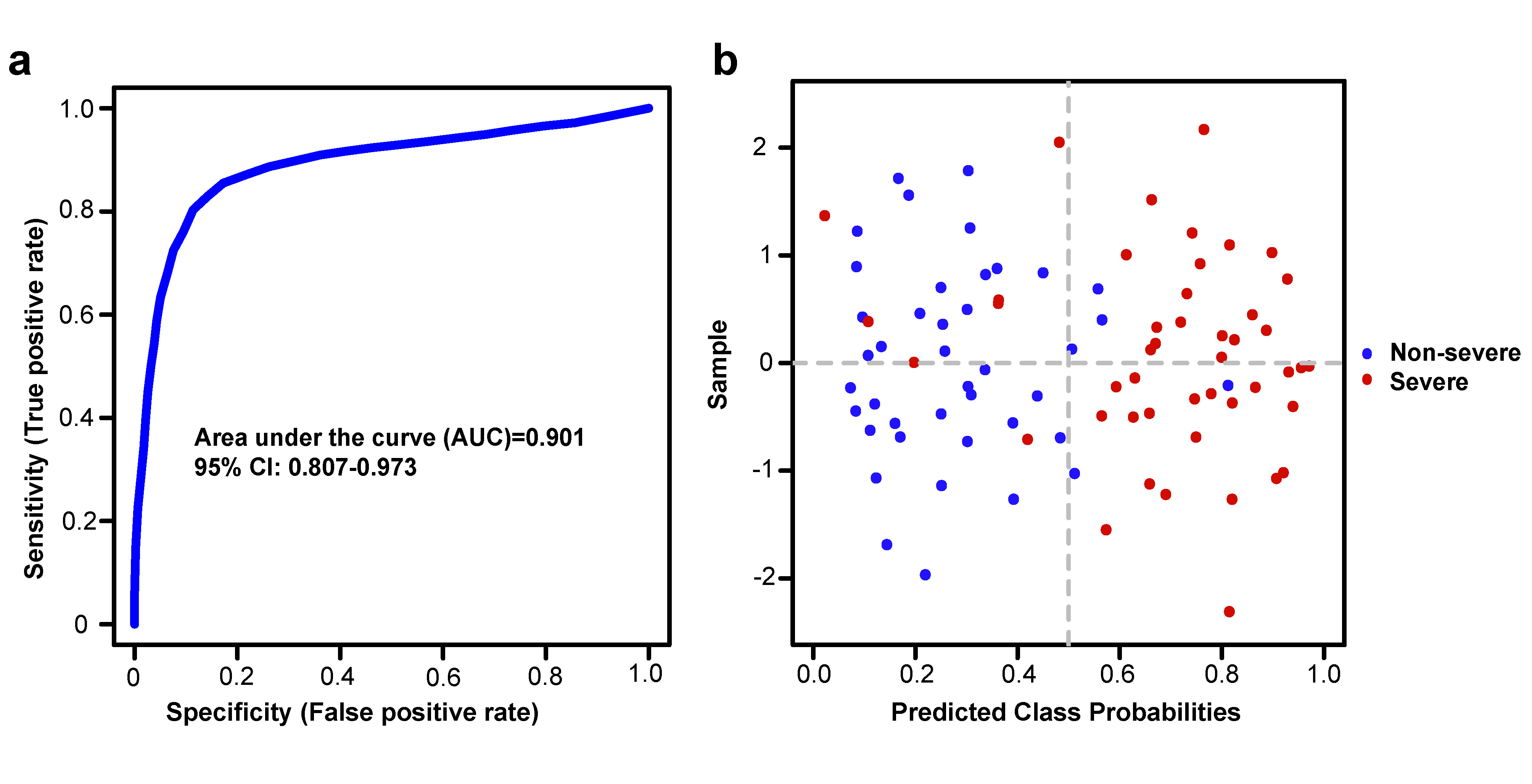


**Fig. S8. Validation performance in public cohort.** (**A**) ROC curve analysis for the predictive power of signatures in public datasets. (**B**) Performance of the model in public datasets.

**Figure. S9.**

**
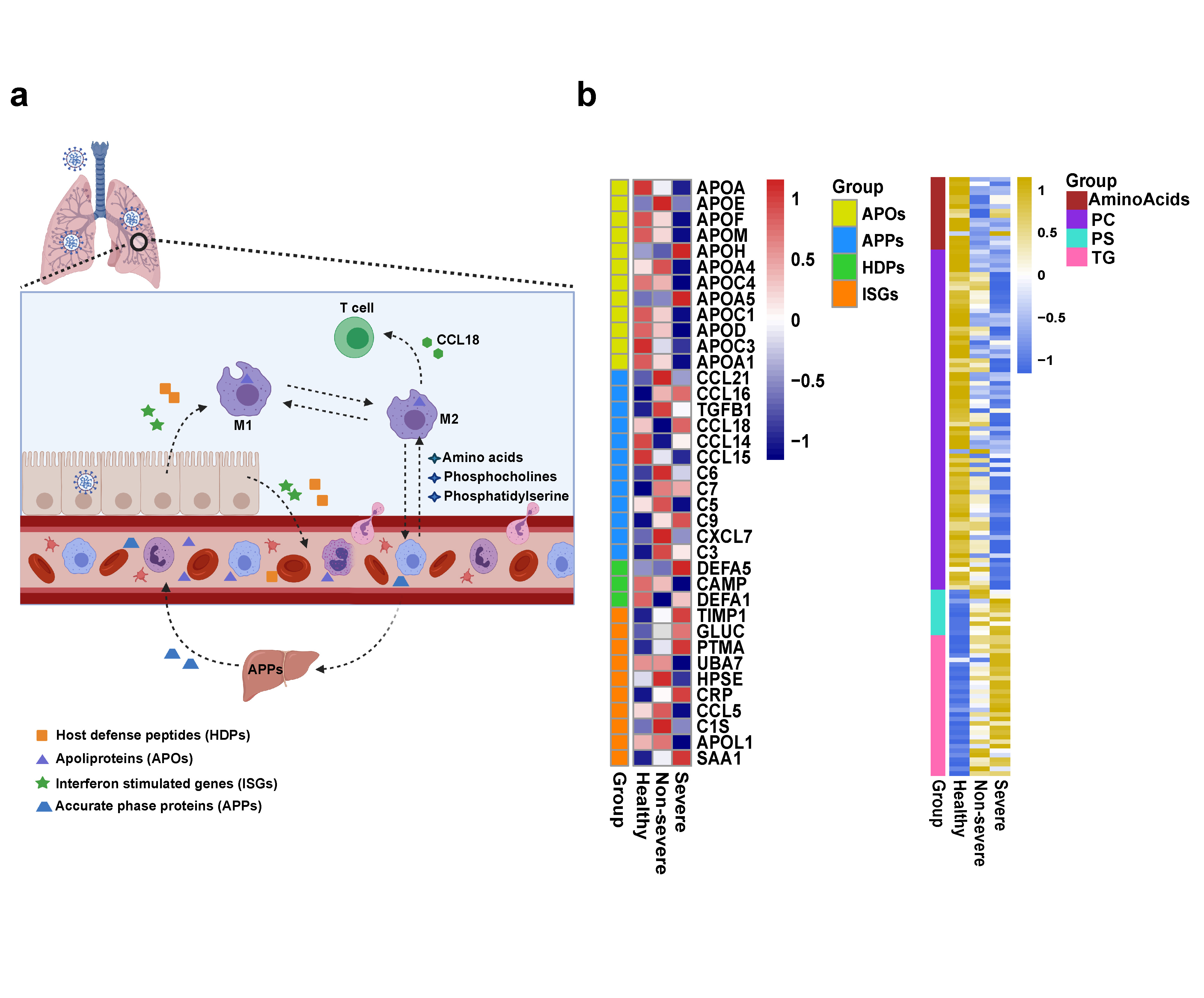
**

**Fig. S9. Molecular mechanism related with SARS-CoV-2 infection.** (**A**) The purposed mechanism of the regulation of proteins, lipids and amino acids during the infection of SARS-CoV-2. (**B**) The abundance difference for proteins, lipids and amino acids participated in the regulation of COVID-19 in non-severe and severe groups compared with healthy controls.

Data S1. (separate file)

Supplementary Dataset 1, available online only

Data S2. (separate file)

Supplementary Dataset 2, available online only
